# The scaffold-level genome sequence of an encrusting sponge, *Halisarca caerulea *Vacelet & Donadey, 1987, and its associated microbial metagenome sequences

**DOI:** 10.12688/wellcomeopenres.24281.1

**Published:** 2025-07-09

**Authors:** Jasper M. de Goeij, Benjamin Mueller, Michelle Achlatis, Sara Campana, Meggie Hudspith, Niklas A. Kornder, Ute Hentschel, Graeme Oatley, Elizabeth Sinclair, Eerik Aunin, Noah Gettle, Camilla Santos, Michael Paulini, Haoyu Niu, Victoria McKenna, Rebecca O’Brien, Wellcome Sanger Institute Tree of Life Management, Samples and Laboratory Team, Wellcome Sanger Institute Scientific Operations: Sequencing Operations, Wellcome Sanger Institute Tree of Life Core Informatics Team, EBI Aquatic Symbiosis Genomics Data Portal Team, Aquatic Symbiosis Genomics Project Leadership

**Affiliations:** 1 Department of Freshwater and Marine Ecology, University of Amsterdam, Amsterdam, The Netherlands; 2 CARMABI Foundation, Willemstad, Curaçao, The Netherlands; 3 Department for Marine Ecology, University of Bremen, Bremen, Germany; 4 GEOMAR Helmholtz Centre for Ocean Research Kiel, Kiel, Germany; 5 Tree of Life, Wellcome Sanger Institute, Hinxton, England, UK

**Keywords:** Halisarca caerulea, encrusting sponge, genome sequence, chromosomal, Chondrillida; metagenome assembly

## Abstract

We present a scaffold-level genome assembly from a *Halisarca caerulea* specimen (encrusting sponge; Porifera; Demospongiae; Chondrillida; Halisarcidae). The genome sequence is 195.70 megabases in span. The mitochondrial genome has also been assembled and is 19.15 kilobases in length. Gene annotation of this assembly on Ensembl identified 26,722 protein-coding genes. The metagenome of the specimen was also assembled and four binned bacterial genomes related to the relevant sponge symbiont clades Alphaproteobacteria bacterium GM7ARS4 and Gammaproteobacteria bacterium *AqS2* ((Tethybacterales) were identified.

## Species taxonomy

Eukaryota; Opisthokonta; Metazoa; Porifera; Demospongiae; Verongimorpha; Chondrillida; Halisarcidae; *Halisarca*; *Halisarca caerulea* Vacelet & Donadey, 1987 (NCBI:txid858189).

## Background

The marine sponge genus *Halisarca* (Dujardin, 1838) has a worldwide distribution and currently includes 22 accepted species ( de Voogd *et al.*, 2024). Species identification in this genus is particularly difficult due to the lack of a mineral (i.e., spicules) or proteinaceous (i.e., spongin fibres) skeleton ( Ereskovsky, 2007; Ereskovsky *et al.*, 2011). Histological, ultrastructural and embryological analyses are needed to identify the species ( Bergquist & Kelly, 2004; Vacelet & Donadey, 1987).

**Figure 1.**
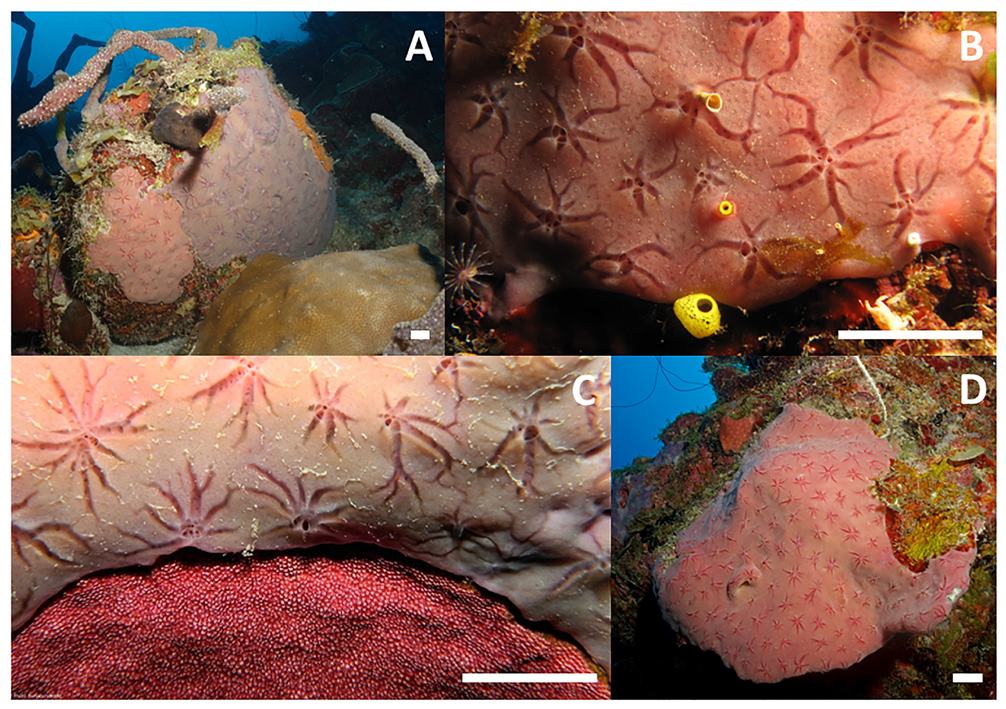
Morphological variations of *Halisarca caerulea* on the reefs of Curaçao. ( **A**) Violet and purple colour morph next to each other. ( **B**) Close up of star-shape canal system surrounding oscula. ( **C**) Shallow water (~10 m) specimen concealed under a coral overhang. ( **D**) Deep water specimen growing openly on the reef at 35 m depth. White scale bar in the lower right corner of each panel represents 2 cm.

The encrusting 1‒3 mm thick “star” sponge *Halisarca caerulea* Vacelet & Donadey 1987 is found on coral reefs ( de Goeij *et al.*, 2008a; Diaz & Rützler, 2009) and in mangroves ( Diaz & Rützler, 2009; Rützler *et al.*, 2000) throughout the wider Caribbean, extending to the east coast of Brazil ( de Moraes, 2011). Individuals can be as small as a few mm ^2^ up to several hundred cm ^2^ in size and range in colour from violet to blue ( Figure 1A). They contain distinctive star-shaped canal systems surrounding their osculum (i.e. outflow opening) ( Figure 1B), distributed evenly every 1‒3 cm across its surface. *Halisarca caerulea* grows on dead coral substrate or mangrove roots from approximately 2 to at least 50 m water depth ( Lesser *et al.*, 2020). In shallow reef areas it is found in low-light, concealed, “cryptic” reef spaces, such as under coral overhangs and in crevices or cavities ( de Goeij *et al.*, 2008a; de Goeij *et al.*, 2013). Below a water depth of around 30 m, *H. caerulea* can also be found in large patches on exposed surfaces of the reef ( Figure 1D).

This sponge is known for its important role in cycling of dissolved organic matter (DOM; the largest available organic resource in the oceans) on coral reefs through the sponge loop ( de Goeij *et al.*, 2013). As one of the four model species described in the original study, *H. caerulea* was found to consume >90 % of its daily carbon diet as DOM ( de Goeij *et al.*, 2008a), of which approximately half is turned over as cellular debris through very rapid cell division and shedding of its filter cells (choanocytes) ( Alexander *et al.*, 2014; De Goeij *et al.*, 2009).

Previously, the classification as either low or high microbial abundance sponge species has been controversial (LMA/HMA) ( Alexander *et al.*, 2014; Maldonado *et al.*, 2012). The thick, collagenous tissue structure resembles that of HMA sponges. However, its physiological performance in terms of uptake and processing rates of particulate and dissolved food sources as well as the excretion of inorganic nutrients resembles that of other LMA species ( Campana *et al.*, 2021; Campana *et al.*, 2024). In combination with the relatively low abundance (10 ^5^‒10 ^6^ prokaryotes per mL), composition, and small cell size of its endosymbiotic prokaryote community, that are more similar to seawater planktonic prokaryotes than to HMA prokaryotic endosymbionts, *H. caerulea* is now considered a LMA species. The prokaryote community of *H. caerulea* is dominated by the phyla Pseudomonadota, Cyanobacteriota, Bacteroidota, and Spirochaetota ( Campana *et al.*, 2024). Within the sponge holobiont (i.e., functional unit consisting of sponge host and associated microbiota), ammonium-oxidising Archaea appear to play a relevant role in internal nitrogen cycling ( Cardoso *et al.*, 2013). Furthermore, carbon fixation and chitin degradation were suggested to support the holobiont metabolism on the shallow and deep reef, respectively ( Lesser *et al.*, 2020).

*Halisarca caerulea* is also a model species in animal-microbe symbiosis studies, for example to show translocation of isotope-tracer labelled food between sponge host and microbial symbionts ( Campana *et al.*, 2021; Campana *et al.*, 2022; de Goeij *et al.*, 2008b; Hudspith *et al.*, 2021). In addition, the species is used as a metazoan model for wound healing and tissue regeneration ( Alexander *et al.*, 2015; Kenny *et al.*, 2018). It is also used as a model for metazoan uptake systems, as its cell kinetics, choanocyte shedding and structure of the choanosome show striking similarities with the human gastrointestinal tract ( de Goeij *et al*. 2009).

The genome of the sponge, *H. caerulea*, was sequenced as part of the Aquatic Symbiosis Genomics (ASG) project, a collaborative effort to sequence 50 sponge species, both marine and freshwater, around the world. *Halisarca caerulea* is the first encrusting and cryptic sponge in this effort. The absence of a skeleton in this species is a unique feature within the Porifera and therefore its genome information adds to the morphological and taxonomic diversity of sponge genomics. Moreover, the family Halisarcidae are proposed to be either highly derived or primitive group of sponges to study the origin of multicellularity ( Maldonado, 2004). Comparative genomics on the recently assembled genome of the sister species *H. dujardinii* will help provide new insights into this family ( Borisenko *et al.*, 2024).

## Genome sequence report

The genome was sequenced from an adult *Halisarca caerulea* ( Figure 1) collected from the southern Caribbean island of Curaçao. A total of 106-fold coverage in Pacific Biosciences single-molecule HiFi long reads was generated. Primary assembly contigs were scaffolded with chromosome conformation Hi-C data. Manual assembly curation corrected 19 missing joins or mis-joins and removed 7 haplotypic duplications, reducing the assembly length by 0.51%.

**Figure 2.**
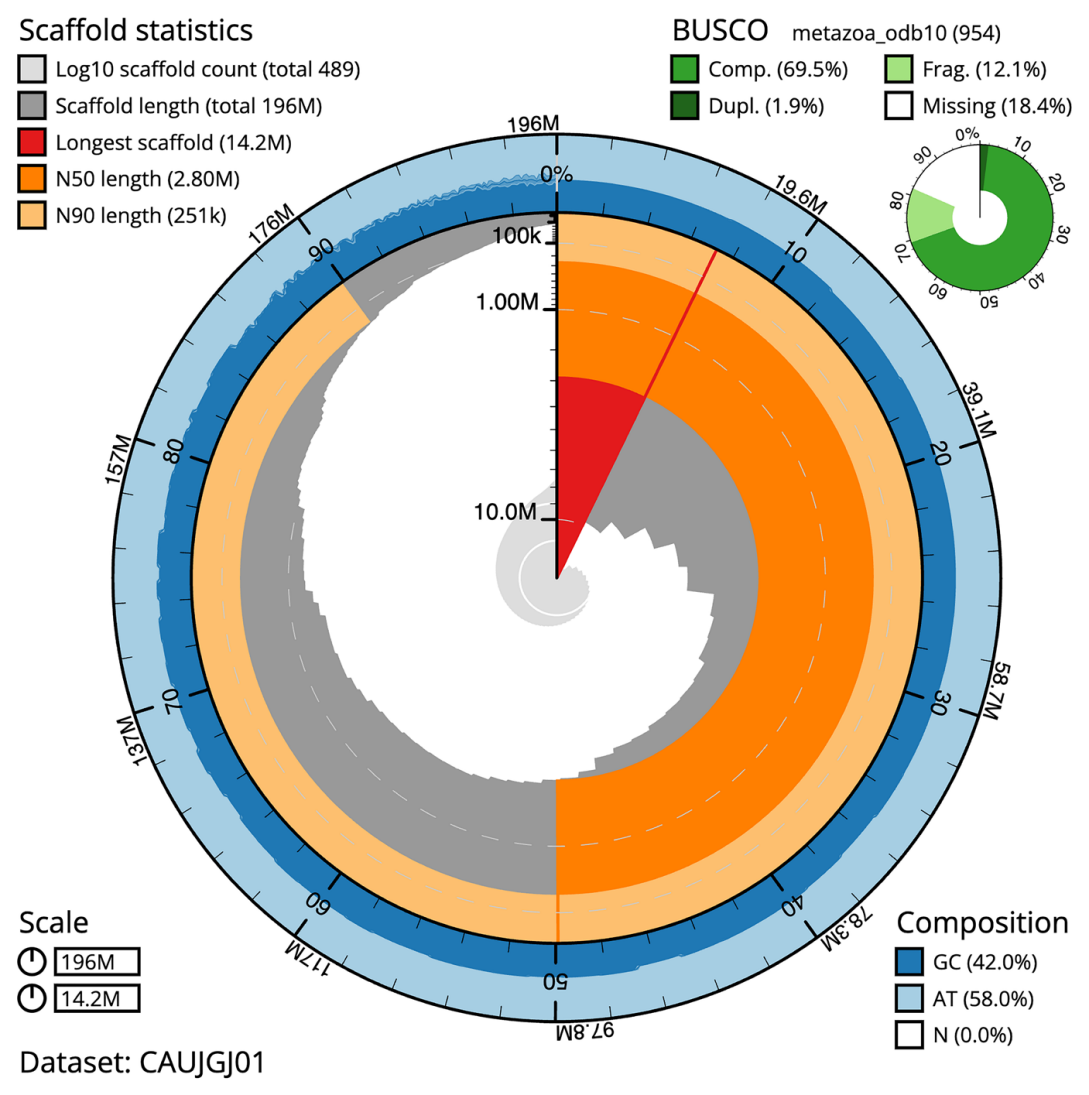
Genome assembly of *Halisarca caerulea*, odHalCaeu1.1: metrics. The BlobToolKit snail plot shows N50 metrics and BUSCO gene completeness. The main plot is divided into 1,000 size-ordered bins around the circumference with each bin representing 0.1% of the 195,692,738 bp assembly. The distribution of sequence lengths is shown in dark grey with the plot radius scaled to the longest sequence present in the assembly (14,164,103 bp, shown in red). Orange and pale-orange arcs show the N50 and N90 sequence lengths (2,795,200 and 251,000 bp), respectively. The pale grey spiral shows the cumulative sequence count on a log scale with white scale lines showing successive orders of magnitude. The blue and pale-blue area around the outside of the plot shows the distribution of GC, AT and N percentages in the same bins as the inner plot. A summary of complete, fragmented, duplicated and missing BUSCO genes in the metazoa_odb10 set is shown in the top right. An interactive version of this figure is available at https://blobtoolkit.genomehubs.org/view/Halisarca%20caerulea/dataset/CAUJGJ01/snail.

**Figure 3.**
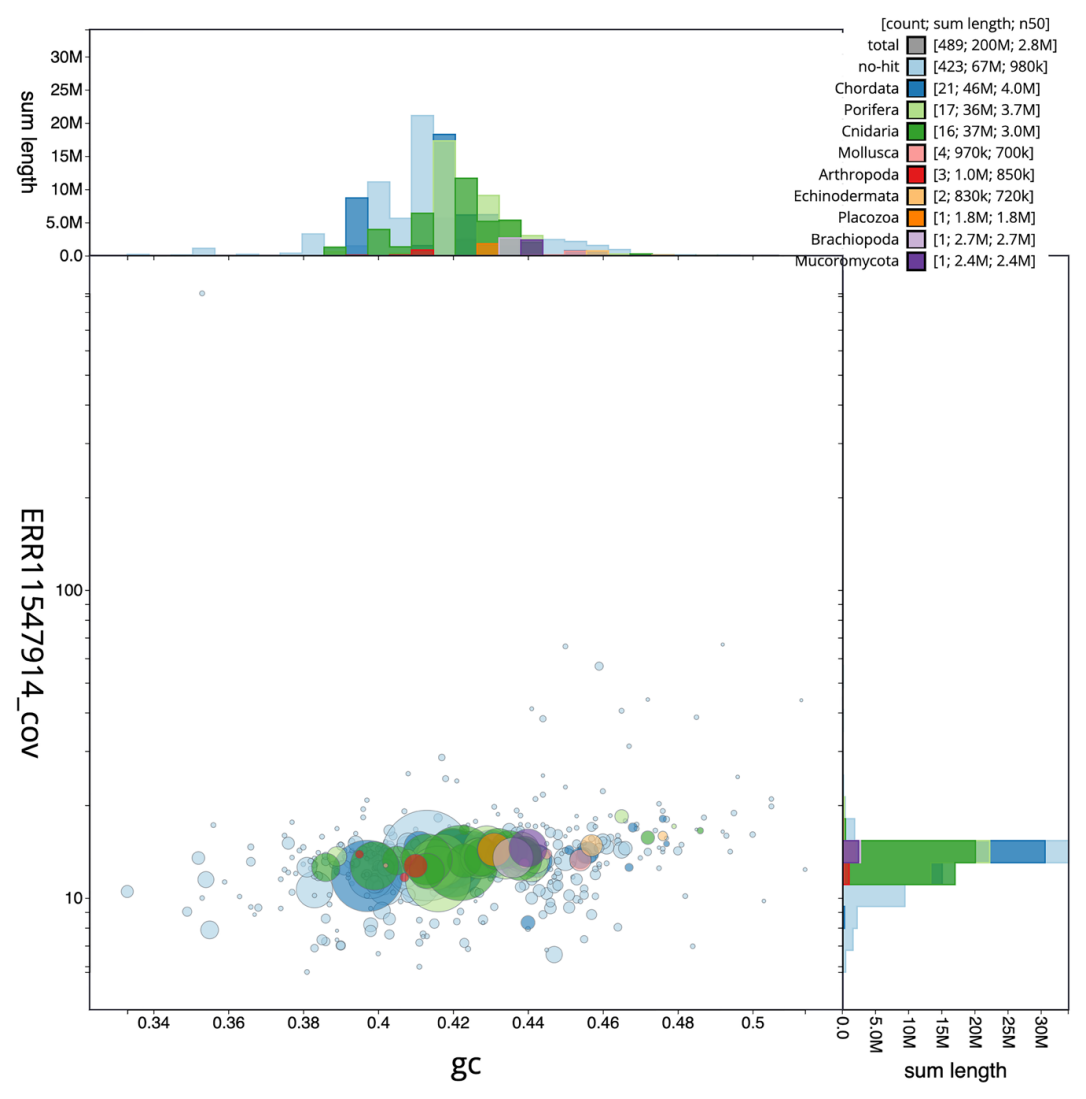
Genome assembly of *Halisarca caerulea*, odHalCaeu1.1: BlobToolKit GC-coverage plot. Scaffolds are coloured by phylum. Circles are sized in proportion to scaffold length. Histograms show the distribution of scaffold length sum along each axis. An interactive version of this figure is available at https://blobtoolkit.genomehubs.org/view/Halisarca%20caerulea/dataset/CAUJGJ01/blob.

**Figure 4.**
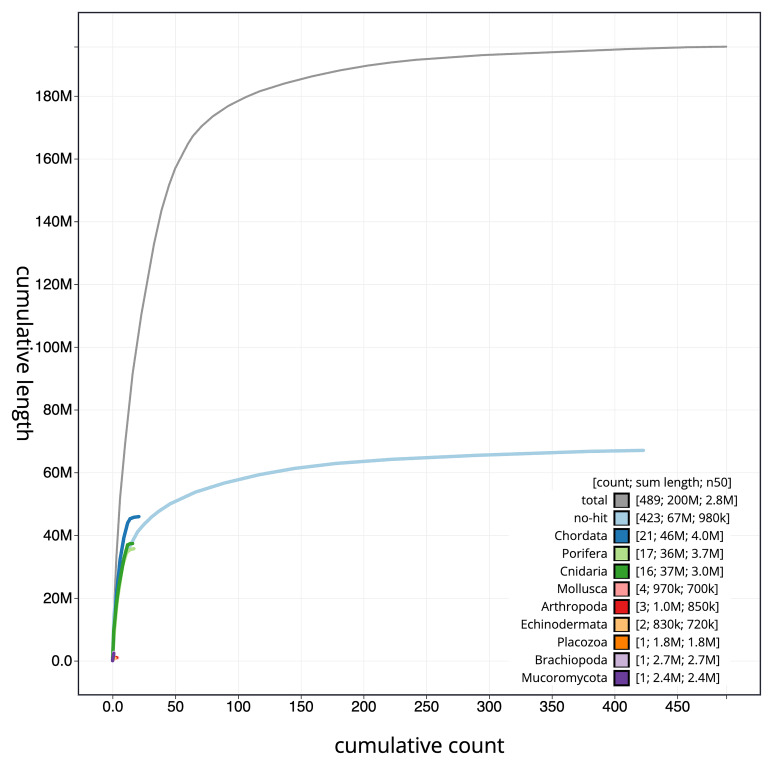
Genome assembly of *Halisarca caerulea*, odHalCaeu1.1: BlobToolKit cumulative sequence plot. The grey line shows cumulative length for all scaffolds. Coloured lines show cumulative lengths of scaffolds assigned to each phylum using the buscogenes taxrule. An interactive version of this figure is available at https://blobtoolkit.genomehubs.org/view/Halisarca%20caerulea/dataset/CAUJGJ01/cumulative.

The final assembly has a total length of 195.70 Mb in 488 sequence scaffolds with a scaffold N50 of 2.8 Mb ( Table 1). The snail plot in Figure 2 provides a summary of the assembly statistics, while the distribution of assembly scaffolds on GC proportion and coverage is shown in Figure 3. The cumulative assembly plot in Figure 4 shows curves for subsets of scaffolds assigned to different phyla. We did not manage to get the assembly to chromosome level, so it is submitted as curated scaffolds. Based on terminal telomers found in the scaffolds, the estimated chromosome number is between 9 and 17. While not fully phased, the assembly deposited is of one haplotype. Contigs corresponding to the second haplotype have also been deposited. The mitochondrial genome was also assembled and can be found as a contig within the multifasta file of the genome submission with GenBank accession OY720623.1.

**Table 1.** Genome data for *Halisarca caerulea*, odHalCaeu1.1.

Project accession data			
Assembly identifier	odHalCaeu1.1		
Species	*Halisarca caerulea*		
Specimen	odHalCaeu1		
NCBI taxonomy ID	858189		
BioProject	PRJEB62893		
BioSample ID	Genome sequencing: SAMEA8576948 Hi-C scaffolding: SAMEA8576954 RNA sequencing: SAMEA8576955		
Isolate information	odHalCaeu1: whole organism (genome sequence) odHalCaeu7: whole organism (Hi-C sequencing) odHalCaeu8: whole organism (RNA sequencing)		
Assembly metrics			
Consensus quality (QV)	50.8		
BUSCO *	C:69.7%[S:67.7%,D:2.0%],F:11.8%,M:18.5%,n:954		
Percentage of assembly mapped to chromosomes	0%		
Sex chromosomes	None		
Organelles	Mitochondrial genome: 19.15 kb		
Sequencing information			
**Platform**	**Run accession**	**Read count**	**Base count (Gb)**
**Hi-C Illumina NovaSeq 6000**	ERR11547915	5.54e+08	83.66
**Hi-C Illumina NovaSeq 6000**	ERR11547916	4.58e+08	69.18
**Hi-C Illumina NovaSeq 6000**	ERR11641141	1.07e+09	161.65
**PacBio Sequel IIe**	ERR11531687	1.70e+06	9.72
**PacBio Sequel IIe**	ERR11531686	1.81e+06	11.98
**RNA Illumina NovaSeq X**	ERR13093635	1.97e+08	29.81
Genome assembly			
Assembly accession	GCA_963170055.1		
*Accession of alternate haplotype*	GCA_963170115.1		
Span (Mb)	195.70		
Number of contigs	901		
Contig N50 length (Mb)	1.7		
Number of scaffolds	488		
Scaffold N50 length (Mb)	2.8		
Longest scaffold (Mb)	14.2		
**Genome annotation**			
Number of protein-coding genes	26,722		
Number of non-coding genes	733		
Number of gene transcripts	39,196		

* BUSCO scores based on the metazoa_odb10 BUSCO set using version 5.4.3. C = complete [S = single copy, D = duplicated], F = fragmented, M = missing, n = number of orthologues in comparison. A full set of BUSCO scores is available at https://blobtoolkit.genomehubs.org/view/Halisarca%20caerulea/dataset/CAUJGJ01/busco.

The estimated Quality Value (QV) of the final assembly is 50.8 with *k*-mer completeness of 99.98%, and the assembly has a BUSCO v5.4.3 completeness of 69.7% (single = 67.7%, duplicated = 2.0%), using the metazoa_odb10 reference set ( *n* = 954).

## Metagenome report

**Figure 5.**
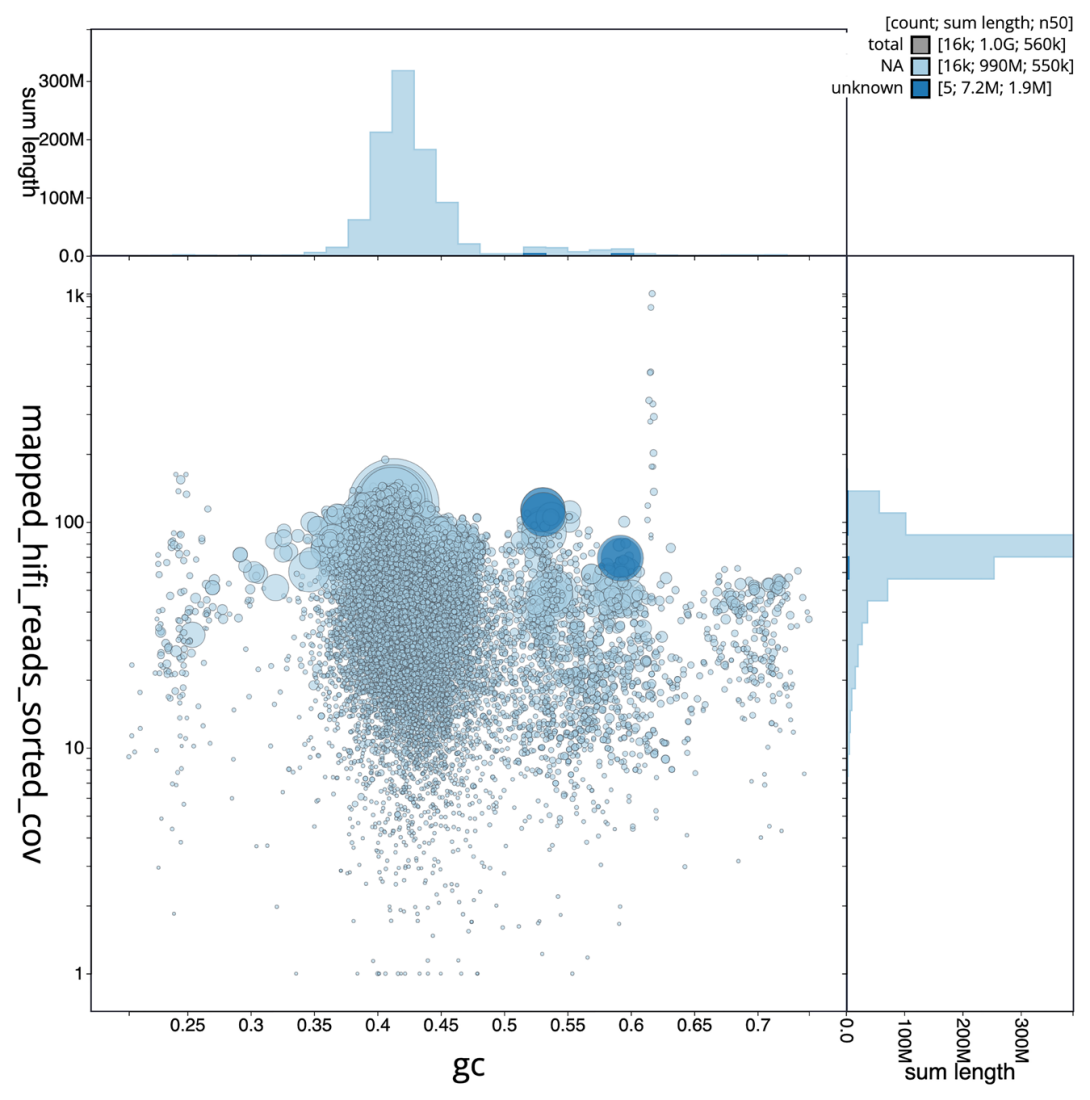
Blob plot of base coverage in mapped against GC proportion for sequences in the *Halisarca caerulea* metagenome. Binned contigs are coloured by family. Circles are sized in proportion to sequence length on a square root scale, ranging from 2,140 to 8,133,556. Histograms show the distribution of sequence length sum along each axis.

Four binned genomes were generated from the metagenome assembly ( Figure 5) of which one was classified as a high-quality metagenome assembled genome (MAG) (see methods). The completeness values for these assemblies range from approximately 71% to 88% with contamination below 2%. For details on binned genomes see Table 2. Interestingly, two binned genomes each fell into the alphaproteobacterial order GM7ARS4 and the gammaproteobacterial family AqS2, MAGs of which have previously been isolated from sponges.

**Table 2.** Quality metrics and taxonomic assignments of the binned metagenomes.

NCBI taxon	Taxid	GTDB taxonomy	Quality	Size (bp)	Contigs	Circular	Mean coverage	Completeness (%)	Contamination (%)
Alphaproteobacteria bacterium	91750	o__GM7ARS4	High	1,869,963	1	Yes	27	87.67	0
Gammaproteobacteria bacterium	86473	f__AqS2	Medium	2,047,616	2	Partial	16.14	88.15	1.22
Gammaproteobacteria bacterium	86473	f__AqS2	Medium	1,470,391	1	No	17.19	71.27	1.22
Alphaproteobacteria bacterium	91750	o__GM7ARS4	High	1,859,046	1	Yes	23.42	87.75	0

## Genome annotation report

The *Halisarca caerulea* genome assembly (GCA_963170055.1) was annotated at the European Bioinformatics Institute (EBI) on Ensembl Rapid Release. The resulting annotation includes 39,196 transcribed mRNAs from 26,722 protein-coding and 733 non-coding genes ( Table 2; https://rapid.ensembl.org/Halisarca_caerulea_GCA_963170055.1/Info/Index). The average transcript length is 5,833.36. There are 1.43 coding transcripts per gene and 5.29 exons per transcript. An alternative annotation is available here: https://github.com/Aquatic-Symbiosis-Genomics-Project/sponge_annotations/tree/main/results/odHalCaeu1.

## Methods

### Sample acquisition

The genome was sequenced from an *H. caerulea* individual (specimen ID GHC0000080, ToLID odHalCaeu1) ( Figure 1) collected on 2021-01-19 by SCUBA diving from reef station “Buoy 1” (latitude 12.12, longitude –68.97) near the Caribbean Research and Management of Biodiversity (CARMABI) marine research station on the southern Caribbean island of Curaçao. A second specimen (specimen ID GHC0000086, ToLID odHalCaeu7) was used for Hi-C sequencing and another for RNA sequencing (specimen ID GHC0000087, ToLID odHalCaeu8). The specimens were collected and identified by Jasper Merijn De Goeij and Benjamin Mueller (University of Amsterdam), and preserved by snap freezing.

### Nucleic acid extraction

The workflow for high molecular weight (HMW) DNA extraction at the Wellcome Sanger Institute (WSI) Tree of Life Core Laboratory includes a sequence of core procedures: sample preparation; sample homogenisation, DNA extraction, fragmentation and clean-up. Protocols developed by the WSI Tree of Life laboratory are publicly available on protocols.io ( Howard *et al.*, 2025). In sample preparation, the odHalCaeu1 sample was weighed and dissected on dry ice ( Jay *et al.*, 2023). Tissue was homogenised using a PowerMasher II tissue disruptor ( Denton *et al.*, 2023). HMW DNA was extracted using the Automated MagAttract v1 protocol ( Sheerin *et al.*, 2023). DNA was sheared into an average fragment size of 12–20 kb in a Megaruptor 3 system ( Todorovic *et al.*, 2023). Sheared DNA was purified by solid-phase reversible immobilisation ( Strickland *et al.*, 2023), using AMPure PB beads to eliminate shorter fragments and concentrate the DNA. The concentration of the sheared and purified DNA was assessed using a Nanodrop spectrophotometer and Qubit Fluorometer and Qubit dsDNA High Sensitivity Assay kit. Fragment size distribution was evaluated by running the sample on the FemtoPulse system.

RNA was extracted from whole organism tissue of odHalCaeu8 in the Tree of Life Laboratory at the WSI using the RNA Extraction: Automated MagMax™ *mir*Vana protocol ( do Amaral *et al.*, 2023). The RNA concentration was assessed using a Nanodrop spectrophotometer and a Qubit Fluorometer using the Qubit RNA Broad-Range Assay kit. Analysis of the integrity of the RNA was done using the Agilent RNA 6000 Pico Kit and Eukaryotic Total RNA assay.

### Sequencing

Pacific Biosciences HiFi circular consensus DNA sequencing libraries were constructed according to the manufacturers’ instructions. Poly(A) RNA-Seq libraries were constructed using the NEB Ultra II RNA Library Prep kit. DNA and RNA sequencing was performed by the Scientific Operations core at the WSI on Pacific Biosciences Sequel IIe (HiFi) and Illumina NovaSeq X (RNA-Seq) instruments. Hi-C data were also generated from whole organism tissue of odHalCaeu7 using the Arima2 kit and sequenced on the Illumina NovaSeq 6000 instrument.

### Genome assembly and curation

Assembly was carried out with Hifiasm ( Cheng *et al.*, 2021) and haplotypic duplication was identified and removed with purge_dups ( Guan *et al.*, 2020). The assembly was then scaffolded with Hi-C data ( Rao *et al.*, 2014) using YaHS ( Zhou *et al.*, 2023). Manual curation was performed using JBrowse2 ( Diesh *et al.*, 2023), HiGlass ( Kerpedjiev *et al.*, 2018) and Pretext ( Harry, 2022). The mitochondrial genome was assembled using MitoHiFi ( Uliano-Silva *et al.*, 2023), which runs MitoFinder ( Allio *et al.*, 2020) and uses these annotations to select the final mitochondrial contig and to ensure the general quality of the sequence.

### Metagenome assembly

The metagenome assembly was generated using Hifiasm (version 0.16.1) and binned using MetaBAT2 ( Kang *et al.*, 2019), MaxBin ( Wu *et al.*, 2014), bin3C ( DeMaere & Darling, 2019), and MetaTOR. The resulting bin sets of each binning algorithm were optimised and refined using MAGScoT ( Rühlemann *et al.*, 2022). PROKKA ( Seemann, 2014) was used to identify tRNAs and rRNAs in each bin, CheckM ( Parks *et al.*, 2015) (checkM_DB release 2015-01-16) was used to assess bin completeness/contamination, and GTDB-TK ( Chaumeil *et al.*, 2022) (GTDB release 214) was used to taxonomically classify bins. Taxonomic replicate bins were identified using dRep ( Olm *et al.*, 2017) with default settings (95% ANI threshold). The final bin set was filtered for bacteria and archaea. All bins were assessed for quality and categorised as metagenome-assembled genomes (MAGs) if they met the following criteria: contamination ≤ 5%, presence of 5S, 16S, and 23S rRNA genes, at least 18 unique tRNAs, and either ≥ 90% completeness or ≥ 50% completeness with fully circularised chromosomes. Bins that did not meet these thresholds, or were identified as taxonomic replicates of MAGs, were retained as ‘binned metagenomes’ provided they had ≥ 50% completeness and ≤ 10% contamination. Software tool versions and sources are given in Table 3.

**Table 3.** Software tools: versions and sources.

Software tool	Version	Source
BEDTools	2.30.0	https://github.com/arq5x/bedtools2
Blast	2.14.0	ftp://ftp.ncbi.nlm.nih.gov/blast/executables/blast+/
BlobToolKit	4.3.7	https://github.com/blobtoolkit/blobtoolkit
BUSCO	5.4.3 and 5.5.0	https://gitlab.com/ezlab/busco
bwa-mem2	2.2.1	https://github.com/bwa-mem2/bwa-mem2
Cooler	0.8.11	https://github.com/open2c/cooler
DIAMOND	2.1.8	https://github.com/bbuchfink/diamond
fasta_windows	0.2.4	https://github.com/tolkit/fasta_windows
FastK	427104ea91c78c3b8b8b49f1a7d6bbeaa869ba1c	https://github.com/thegenemyers/FASTK
GoaT CLI	0.2.5	https://github.com/genomehubs/goat-cli
Hifiasm	0.15.3-r339	https://github.com/chhylp123/hifiasm
HiGlass	44086069ee7d4d3f6f3f0012569789ec138f42b84 aa44357826c0b6753eb28de	https://github.com/higlass/higlass
MerquryFK	d00d98157618f4e8d1a9190026b19b471055b22e	https://github.com/thegenemyers/MERQURY.FK
MitoHiFi	2	https://github.com/marcelauliano/MitoHiFi
MultiQC	1.14, 1.17, and 1.18	https://github.com/MultiQC/MultiQC
Nextflow	23.04.0-5857	https://github.com/nextflow-io/nextflow
PretextView	0.2.5	https://github.com/wtsi-hpag/PretextView
purge_dups	1.2.3	https://github.com/dfguan/purge_dups
samtools	1.16.1, 1.17, and 1.18	https://github.com/samtools/samtools
Seqtk	1.3	https://github.com/lh3/seqtk
Singularity	3.9.0	https://github.com/sylabs/singularity
TreeVal	1.0.0	https://github.com/sanger-tol/treeval
YaHS	1.1a.2	https://github.com/c-zhou/yahs

### Evaluation of the final assembly

The MerquryFK tool ( Rhie *et al.*, 2020), run within a Singularity container ( Kurtzer *et al.*, 2017), was used to evaluate *k*-mer completeness and assembly quality for the primary and alternate haplotypes, using the *k*-mer databases ( *k* = 31) computed before genome assembly. The analysis outputs included the assembly QV consensus quality value. A Hi-C map for the final host and eukaryote cobiont assemblies were produced using bwa-mem2 ( Vasimuddin *et al.*, 2019) in the Cooler file format ( Abdennur & Mirny, 2020). The genome was also analysed within the BlobToolKit environment ( Challis *et al.*, 2020) and BUSCO scores ( Manni *et al.*, 2021) were calculated.

Table 3 contains a list of relevant software tool versions and sources.

### Genome annotation

The Ensembl Genebuild annotation system ( Aken *et al.*, 2016) was used to generate annotation for the *Halisarca caerulea* assembly (GCA_963170055.1) in Ensembl Rapid Release at the EBI. Annotation was created primarily through alignment of transcriptomic data to the genome, with gap filling via protein-to-genome alignments of a select set of proteins from UniProt ( UniProt Consortium, 2019).

### Wellcome Sanger Institute – Legal and Governance

The materials that have contributed to this genome note have been supplied by a Tree of Life collaborator. The Wellcome Sanger Institute employs a process whereby due diligence is carried out proportionate to the nature of the materials themselves, and the circumstances under which they have been/are to be collected and provided for use. The purpose of this is to address and mitigate any potential legal and/or ethical implications of receipt and use of the materials as part of the research project, and to ensure that in doing so we align with best practice wherever possible. The overarching areas of consideration are:

•    Ethical review of provenance and sourcing of the material

•    Legality of collection, transfer and use (national and international)

Each transfer of samples is undertaken according to a Research Collaboration Agreement or Material Transfer Agreement entered into by the Tree of Life collaborator, Genome Research Limited (operating as the Wellcome Sanger Institute) and in some circumstances other Tree of Life collaborators.

## Data Availability

European Nucleotide Archive: Halisarca caerulea. Accession number PRJEB62893; https://identifiers.org/ena.embl/PRJEB62893. The genome sequence is released openly for reuse. The *Halisarca caerulea* genome sequencing initiative is part of the Aquatics Symbiosis Genomics (ASG) project. All raw sequence data and the assembly have been deposited in INSDC databases. The genome will be annotated using available RNA-Seq data and presented through the Ensembl pipeline at the European Bioinformatics Institute. Raw data and assembly accession identifiers are reported in Table 1 and Table 2.

## Author information

Members of the Wellcome Sanger Institute Tree of Life Management, Samples and Laboratory Team are listed here: https://doi.org/10.5281/zenodo.10066175.

Members of the Wellcome Sanger Institute Scientific Operations: Sequencing Operations are listed here: https://doi.org/10.5281/zenodo.10043364.

Members of the Wellcome Sanger Institute Tree of Life Core Informatics team are listed here: https://doi.org/10.5281/zenodo.10066637.

Members of the European Bioinformatics Institute ASG Data Portal team are listed here: https://doi.org//10.5281/zenodo.10076466.

Members of the Wellcome Sanger Institute/Aquatic Symbiosis Genomics Project Leadership are listed here: https://doi.org/10.5281/zenodo.10184833.
