# Supplementary material for: The scaffold-level genome sequence of an encrusting sponge, Halisarca caerulea Vacelet & Donadey, 1987, and its associated microbial metagenome sequences
Source: Wellcome Open Res. 2025 Jul 9;10:344. [Version 1] doi: 10.12688/wellcomeopenres.24281.1 (PMC12391866; doi:10.12688/wellcomeopenres.24281.1)
Supplement: Peer review history

# Reviewer report: Reviewer response for version 1

**DOI:** 10.21956/wellcomeopenres.26779.r127598

**Published:** 2025-08-27

**Authors:** Rodrigo Costa

**Affiliations:** 1 Universidade de Lisboa, Lisbon, Lisbon, Portugal

De Goeij et al report on the genome sequence and associated microbial metagenome of the marine sponge *Halisarca caerulea*. This species is a valuable resource within the Porifera owing to its unusual absence of mineral and proteinaceous skeletal structures, typical of other marine sponges. The genome could not be assembled at the chromosomal level and just four bacterial MAGs are reported, suggesting limitations in the retrieval of contig/scaffolds with high coverage. Nevertheless, making these datasets available, including RNA-seq data, will benefit the scientific community interested in sponge physiology, evolution, and symbiosis at large.

**General comments:**

 Because of the stringency involved in the selection of MAGs to be reported, requiring MAGs to present rRNA genes, only four MAGs passed the quality thresholds used and, in some instances of the manuscript (particularly, in the abstract), this gives the impression that the *H. caerulea* microbiome is rather poor and constituted by a few bacterial species. In the minor/specific comments below, I suggest a few interventions to fix this issue, including modifications to Figure 5 that are needed to provide a more accurate perspective of microbiome diversity in *H. caerulea*. Then authors should ideally discuss, in the text (one-two sentences is enough), the extent to which microbial diversity observed in this study (including MAGs and metagenome contigs) compares with previous reports of microbial diversity in *H. caerulea *(e.g. Campana et al. 2024).

 - Please revise the ms. throughout to highlight (e.g., using italics) scientific names with standing in nomenclature. This is valid for the sponge genus Halisarca, for the sponge species *H. caerulea*, and for all prokaryotic taxon names (from phylum to species) mentioned in the text.

**Specific comments:**

**ABSTRACT:**

 “four binned bacterial genomes… were identified”.

 In the context of the abstract, this gives the impression that the *H. caerulea* microbiome is composed of two species only. I suggest broadening this perspective here and adding a note of the family-level taxonomy (or even class or phylum level) of the *H. caerulea* microbiome, according to the data provided in Figure 5 (more on this below).

**BACKGROUND**

**2 ^nd^ paragraph:**

 There is no mention of Figure 1C. Please correct this so that all items A through D are cited in the text in sequential order. Alternatively, simply refer to "Figure 1" in all instances where this figure is mentioned throughout the manuscript.

**4 ^th^ paragraph, first sentence:**

 Replace “classification” with “classification of *H. caerulea*” for better readability.

**4 ^th^ paragraph:**

  “…by the phyla Pseudomonadota… and Spirochaeota (Campana et al., 2024)”.

 Considering that only two MAGs/phylotypes are reported, one could disclose a bit more precisely, here, what is the species-level (ASVs, single-species genome bins/MAGs etc), and family-level diversity known for this sponge. Then, in the “metagenome report” section, a note could be added on how microbiome diversity reported in this study compares with that reported by Campana et al and/or other studies.

**6 ^th^ paragraph**

 “primitive”: most likely “basal” and early-diverging” are better word choices.

**GENOME SEQUENCE REPORT**

**1 ^st^ paragraph, 1 ^st^ line:**

 Replace “adult” with “adult specimen of” for better readability.

**METAGENOME REPORT**

**1 ^st^ paragraph, “(Figure 5)”: **From Figure 5, as currently presented, it is not possible to identify the four binned genomes listed in Table 2. Please adjust the Figure accordingly or rephrase the sentence so it fits the display item(s) you are referring to.

**Figure 5 (continued): **There is no legend provided as an inlet within the figure to allow the visualization of different families (based on colours).

 Where are the four assembled MAGs? Can they be located on this landscape (see comment above on this)?

 As mentioned in my comment to the abstract, this figure can help discuss microbial diversity in *H. caerulea* in a broader context, beyond the two phylotypes represented by the MAGs, and in line with Campana et al (2024) regarding what is known in terms of microbial diversity in this sponge. Are your results similar to those reported by Campana et al? One or two sentences on the matter will be enough, in my opinion.

**Legend to Figure 5:** Add unit after “8,133,556”.

**GENOME ANNOTATION REPORT**

 Add unit (bp) after “5,833.36”

**METHODS – Sample acquisition**

 “A second specimen…was used…for RNA sequencing”.

 Perhaps this should be better contextualized in the genome report section, for improved readability. Only here the reader learns that RNA seq was employed. As RNA seq is apparently less frequent among genome notes, mentioning that dedicated RNA seq was performed while reporting the data in the sections above can be helpful.

**Caption to Table 2.** Replace “metagenomes” with “MAGs” or “genomes”. You are listing individual genomes here.

Are sufficient details of methods and materials provided to allow replication by others?

Yes

Is the rationale for creating the dataset(s) clearly described?

Yes

Are the datasets clearly presented in a useable and accessible format?

Partly

Are the protocols appropriate and is the work technically sound?

Yes

Reviewer Expertise:

Metagenomics, molecular microbial ecology, symbioses, microbiomes, blue biotechnology

I confirm that I have read this submission and believe that I have an appropriate level of expertise to confirm that it is of an acceptable scientific standard.

# Reviewer report: Reviewer response for version 1

**DOI:** 10.21956/wellcomeopenres.26779.r127599

**Published:** 2025-08-27

**Authors:** Matsapume Detcharoen

**Affiliations:** 1 Prince of Songkla University, Hat Yai, Thailand

This study reports a scaffold-level genome assembly of the sponge *Halisarca caerulea*. The genome is 195.70 Mb in length and distributed over 488 scaffolds. Genome annotation identified 26,722 protein-coding genes and 733 non-coding genes. Metagenome analysis classified four binned bacterial genomes related to typical sponge symbionts. Two of these belonged to the alphaproteobacterial order GM7ARS4, and two of the gammaproteobacterial family AqS2. These microbial clades are considered typical sponge symbionts and have previously been identified in other sponge species. The authors acknowledge the absence of a chromosome-level assembly, achieving one would significantly improve genome completeness and contiguity. My comments would be consider a more in-depth analysis of the metagenome which can provide a fuller understanding of the diverse microbial community associated with *H. caerulea* (e.g., functional annotation).

Are sufficient details of methods and materials provided to allow replication by others?

Yes

Is the rationale for creating the dataset(s) clearly described?

Yes

Are the datasets clearly presented in a useable and accessible format?

Yes

Are the protocols appropriate and is the work technically sound?

Yes

Reviewer Expertise:

Molecular ecology and symbiosis

I confirm that I have read this submission and believe that I have an appropriate level of expertise to confirm that it is of an acceptable scientific standard.
